# *In Silico* Prediction of *BRCA1* and *BRCA2* Variants with Conflicting Clinical Interpretation in a Cohort of Breast Cancer Patients

**DOI:** 10.3390/genes15070943

**Published:** 2024-07-18

**Authors:** Stefania Stella, Silvia Rita Vitale, Michele Massimino, Federica Martorana, Irene Tornabene, Cristina Tomarchio, Melissa Drago, Giuliana Pavone, Cristina Gorgone, Chiara Barone, Sebastiano Bianca, Livia Manzella

**Affiliations:** 1 Department of Clinical and Experimental Medicine, University of Catania, 95123 Catania, Italy; federica.martorana@unict.it (F.M.); irenetornabene@gmail.com (I.T.); cristina.tomarchio@hotmail.it (C.T.); dragomelissa23@gmail.com (M.D.); giuliana.pavone@humanitascatania.it (G.P.); manzella@unict.it (L.M.); 2 Center of Experimental Oncology and Hematology, A.O.U. Policlinico “G. Rodolico—San Marco”, 95123 Catania, Italy; silviarita.vitale@gmail.com (S.R.V.); michedot@yahoo.it (M.M.); 3 Department of General Surgery and Medical-Surgical Specialties, University of Catania, 95123 Catania, Italy; 4 Division of Pathology, Humanitas Istituto Clinico Catanese, 95045 Catania, Italy; 5 Medical Oncology Unit, Humanitas Istituto Clinico Catanese, 95045 Catania, Italy; 6 Istituto Oncologico del Mediterraneo—IOM, 95029 Catania, Italy; cristinagorgone@gmail.com; 7 Medical Genetics, ASP, 96100 Siracusa, Italy; ambulatoriogenetica@gmail.com; 8 Medical Genetics, ARNAS Garibaldi, 95123 Catania, Italy; geneticamedica@arnasgaribaldi.it

**Keywords:** *BRCA1*, *BRCA2*, genetic test, VUS, conflicting interpretation of pathogenicity (CIP), NGS, breast cancer, *in silico* tools, PolyPhen-2, SIFT, mutation taster, PROVEAN

## Abstract

Germline *BRCA1/2* alteration has been linked to an increased risk of hereditary breast and ovarian cancer syndromes. As a result, genetic testing, based on NGS, allows us to identify a high number of variants of uncertain significance (VUS) or conflicting interpretation of pathogenicity (CIP) variants. The identification of CIP/VUS is often considered inconclusive and clinically not actionable for the patients’ and unaffected carriers’ management. In this context, their assessment and classification remain a significant challenge. The aim of the study was to investigate whether the *in silico* prediction tools (PolyPhen-2, SIFT, Mutation Taster and PROVEAN) could predict the potential clinical impact and significance of *BRCA1/2* CIP/VUS alterations, eventually impacting the clinical management of Breast Cancer subjects. In a cohort of 860 BC patients, 10.6% harbored *BRCA1* or *BRCA2* CIP/VUS alterations, mostly observed in *BRCA2* sequences (85%). Among them, forty-two out of fifty-five alterations were predicted as damaging, with at least one *in silico* that used tools. Prediction agreement of the four tools was achieved in 45.5% of patients. Moreover, the highest consensus was obtained in twelve out of forty-two (28.6%) mutations by considering three out of four *in silico* algorithms. The use of prediction tools may help to identify variants with a potentially damaging effect. The lack of substantial agreement between the different algorithms suggests that the bioinformatic approaches should be combined with the personal and family history of the cancer patients.

## 1. Introduction

Breast cancer (BC) represents the most frequent malignancy worldwide and the most lethal in women, with 2 million new cases diagnosed each year [1,2,3]. In addition to the biological features, including BC subtypes, tumor grading and other transversal biomarkers that directly and independently correlate with tumor aggressiveness, inherited genetic alteration plays a role in BC hereditary predisposition [4]. Different epidemiological studies identified a set of eight genes (*ATM*, *BARD1*, *BRCA1*, *BRCA2*, *CHEK2*, *PALB2*, *RAD51C* and *RAD51D*) mostly responsible for hereditary BC. Among these, the two main susceptibility genes correlated with a higher risk to develop breast and ovarian carcinoma are *BRCA1* and *BRCA2* [5,6,7,8].

Germline *BRCA1* and *BRCA2* alterations increase the probability to develop BC and other tumor types, including ovarian, pancreatic, prostate, colorectal cancer and melanoma [9]. Over the years, the availability of next generation sequencing (NGS) technologies led to an increase of *BRCA1* and *BRCA2* genetic testing requests to improve diagnoses, prognostic information, research and clinical practice. The currently accepted method for the *BRCA1* and *BRCA2* variants’ classification is based on the Evidence-based Network for the Interpretation of Germline Mutant Alleles (ENIGMA) consortium classification, supported by the ClinVar database, according to the International Agency for Research on Cancer (IARC)’s recommendations [10,11]. This classification system includes five classes, as follows: (i) benign (class I); (ii) likely benign (class II); (iii) variant of uncertain significance (VUS, class III); (iv) likely pathogenic (class IV) and (v) pathogenic (PV, class V). A VUS or conflicting interpretation of pathogenicity (CIP) variant consists of an alteration in the gene sequence, with unknown consequences on the function of the gene product or on the potential risk for disease development. The identification of VUS is evaluated as inconclusive and clinically not actionable for the patients’ and unaffected carriers’ management.

To date, the frequency of VUS reporting is about 10–20% in women who have undergone *BRCA* analysis [12,13]. This frequency depends on the testing prevalence and/or population ancestry [14]. Several studies reported a VUS frequency of 21% in the African-American population, 5–6% in the European ancestry population and up to 15% in European subjects [15,16,17].

The significant rate of CIP/VUS retrieval furthers a claim for an alternative approach of their annotation or re-classification as benign or pathogenic alterations. With the advances of bioinformatics biology, different groups have improved high-throughput pipelines and *in silico* tools to identify functionally germline CIP/VUS alterations [18,19,20,21]. Among them, Polymorphism Phenotyping v.2 (PolyPhen-2) [22] and Sorting Intolerant From Tolerant (SIFT) [23] are two algorithms used to assess the functional impact of missense alterations, whereas the Mutation Taster (MT) [24] and Protein Variation Effect Analyzer (PROVEAN) [25] can be utilized also to improve potential deleterious effects of synonymous or intronic and indel mutations.

In this study, we evaluated the *in silico* predictions of *BRCA1* and *BRCA2* CIP/VUS alterations in a cohort of BC patients, according to the ENIGMA or ClinVar database, using PolyPhen-2, SIFT, MT and PROVEAN tools. By doing so, we investigated whether these algorithms could predict the clinical effect and significance of CIP/VUS alterations, eventually impacting the clinical management of subjects diagnosed with BC.

## 2. Patients and Methods

### 2.1. Study Population

A retrospective collection of molecular data from a total of 1027 patients with breast and ovarian cancer, melanoma, pancreatic tumor or prostate carcinoma was carried out at the “Center of Experimental Oncology and Hematology” of the Hospital Policlinico “G. Rodolico—San Marco” of Catania from January 2017 to May 2023. The patients were referred to our Molecular Diagnostics Laboratory for *BRCA1* and *BRCA2* genetic testing. The present study was conducted in accordance with the Declaration of Helsinki and in accordance with the local legislation and institutional review board. All participants provided their written informed consent before undergoing molecular analysis and to participate in this study.

Oncogenetic counselling was carried out for each subject by a multidisciplinary team including an oncologist, a geneticist and a psychologist before performing *BRCA1* and *BRCA2* mutational analysis, with the purpose of identifying patients at high risk of harboring a PV in *BRCA1* and/or *BRCA2* genes. Patient selection was performed according to the Italian Association of Medical Oncology (AIOM) guidelines (https://www.aiom.it/linee-guida-aiom-2023-carcinoma-mammario-in-stadio-precoce/, accessed on 11 July 2024) and the Sicily local recommendations, Percorso Diagnostico Terapeutico e Assistenziale (PDTA) relativo alla Sindrome dei tumori eredo-familiari della mammella e/o dell’ovaio (http://pti.regione.sicilia.it/portal/page/portal/PIR_PORTALE/PIR_LaStrutturaRegionale/PIR_AssessoratoSalute/PIR_Infoedocumenti/PIR_DecretiAssessratoSalute/PIR_DecretiAssessoriali/PIR_DecretiAssessorialianno2019/Allegato%20al%20D.A.%20n.32.pdf. The latest version released on 11 July 2024.

### 2.2. DNA Extraction from Patients

Each patient provided 20 milliliters (mL) of peripheral blood within a single blood draw, which was collected into EDTA tubes (BD Biosciences, Franklin Lakes, NJ, USA). After the blood draw, fenomic DNA was isolated from 0.7 mL of whole blood samples using the Qiasymphony DSP DNA Midi kit Isolation Kit (QIAGEN, Hilden, Italy), and quantified using a Qubit^®^3.0 or Qubit^®^3.0 fluorometer (Thermofisher Scientific, Waltham, MA, USA), according to the manufacturer’s instructions.

### 2.3. Next Generation Sequencing Analysis for BRCA1/2 Genes

Target enrichment and library preparation was performed using an Oncomine™ BRCA Research Assay Chef. After the preparation, the samples were loaded into an Ion AmpliSeq™ Chef Reagents DL8 cartridge for automated libraries preparation, as previously published [7]. The kit contains two multiplex PCR primer pools, proficient to study all *BRCA1* (NM_007300.3) and *BRCA2* (NM_000059.3) genes. In brief, 10 ng of each DNA sample were placed in the barcode plate for library preparation. The plate, with all reagents and consumables, was loaded on the Ion Chef™ Instrument. An automated library preparation and pooling of barcoded sample libraries was then performed on the Ion Chef™ Instrument. The quantity of pooled libraries was evaluated using a Qubit^®^3.0 or 4.0 fluorometer. Lastly, the pooled libraries were mixed in an equimolar ratio in the Ion Chef™ Library Sample Tube and loaded onto the Ion Chef™ Instrument. The sequencing was performed with an Ion 510 or 520 Chip, using an Ion Gene Studio S5 Plus System instrument (Thermofisher Scientific, Waltham, MA, USA). Analysis of data was performed with Amplicon Suite (SmartSeq s.r.l., Alessandria, Italy) and Ion Reporter Software (v. 5.20.2.0).

### 2.4. Data Analysis and Genetic Classification

All alterations’ nomenclature followed current guidelines of the Human Genome Variation Society, available online (HGVS, http://www.hgvs.org/mutnomen, accessed on 11 July 2024). The clinical significance of *BRCA1* and *BRCA2* alterations was characterized using the classification of the consortium ENIGMA (Evidence-based Network for the Interpretation of Germline Mutant Alleles, https://enigmaconsortium.org/, accessed on 11 July 2024), and by consulting several databanks, such as ClinVar, ARUP, BRCA Exchange, IARC_LOVD and UMD. The classification includes the following five distinct classes of risk: benign (class I), likely benign (class II), variant of uncertain significance (VUS, class III), likely pathogenic (class IV) and pathogenic (class V). The alterations were classified with a conflicting interpretation of pathogenicity, and variants of uncertain significance were defined as CIP/VUS, respectively. The effect of alterations on protein structure and function was also analyzed by VarSome, an informatic tool that allows access to about 30 databases, as previously described [7,26].

### 2.5. Sanger Sequencing

The presence of each CIP/VUS mutation was verified by Sanger sequencing. A specific primer forward and reverse were designed for each of the detected alterations by using the *BRCA1* and *BRCA2* gene reference sequences (NG_005905.2, NM_007294.3 and NG_012772.3, NM_000059.3, respectively). After, a PCR specific to the target sequence was performed, followed by Sanger sequencing.

### 2.6. In Silico Analysis

To attribute a potential clinical significance to each CIP/VUS, the following *in silico* prediction tools were used: Protein Variation Effect Analyzer (PROVEAN—http://provean.jcvi.org/index.php, accessed on 11 July 2024); Sorting Intolerant From Tolerant (SIFT—https://sift.bii.a-star.edu.sg/, accessed on 11 July 2024), Mutation Taster (https://www.mutationtaster.org, accessed on 11 July 2024) and Polymorphism Phenotyping v.2 (PolyPhen-2-http://genetics.bwh.harvard.edu/pph2/, accessed on 11 July 2024), as previously reported [27,28].

PROVEAN is a bioinformatic tool for the screening of alterations in order to determine an amino acid substitution, nonsynonymous or indel variants that are predicted to have an impact on protein function.

PolyPhen-2 is a tool which predicts the effect of the amino acid change on a human protein function and structure. The algorithm identifies protein ID numbers and entry names using the UniProtKB database. The tool generates a report in which the alteration may be classified into three categories, as follows: benign, possibly damaging and probably damaging. A result of “benign” (score ≤ 0.5) suggests that the mutation is not likely to affect protein function. A result of “possibly damaging” (0.5 < score ≤ 0.9) points to a possible effect on protein function. A result of “probably damaging” (score > 0.9) indicates that the alteration is likely to impact protein function. The algorithm uses an amino acid alteration or a protein sequence as input, then performs a BLAST search to recognize homologous sequences and generates scores. The variants are classified into a functional category, either deleterious or neutral, based on a pre-set threshold, with a default threshold value fixed at −2.5. Therefore, variants with scores below this threshold are categorized as deleterious.

MutationTaster is an online tool able to investigate the potential effect of DNA sequence variants on the gene products. The algorithm works on both protein and DNA level by testing substitutions of single amino acids, as well as synonymous or intronic variant. The alteration is predicted as one of four possible types: (i) disease causing (probably deleterious), (ii) disease causing automatic (variation known to be deleterious), (iii) polymorphism (probably neutral) and (iv) polymorphism automatic (known to be neutral).

SIFT is a bioinformatics program that predicts whether an amino acid alteration may have an impact on protein function. The software analyzes nonsynonymous polymorphisms and missense mutations based on sequence homology and the physical properties of amino acids. Therefore, SIFT chooses related proteins and elaborates an alignment of these proteins with the query. Finally, the software estimates the probability that the toleration of an amino acid at a position is conditional on the most frequent amino acid being tolerated. The score can range from 0 to 1, and substitutions with scores < 0.05 are considered deleterious.

The CIP/VUS alterations predicted as “possibly/probably damaging/intolerant” were classified as a “damaging” variant, whereas the mutations predicted as “benign moderate or strong/tolerant” were classified as a “neutral” variant.

## 3. Results

### 3.1. Population Characteristics

**Figure 1:**
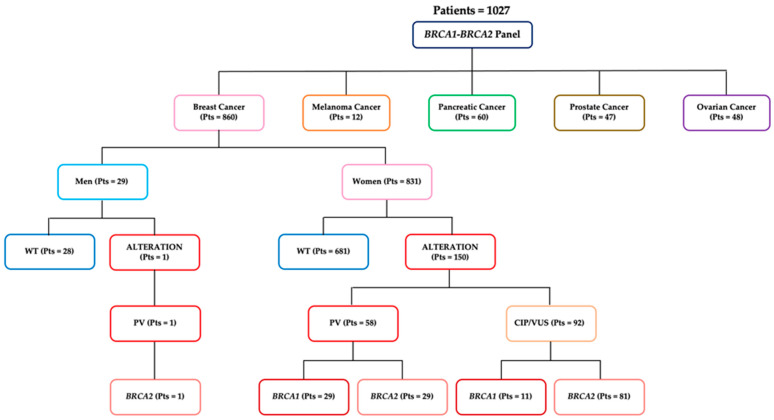
Overview of the study. *BRCA1* and *BRCA2* gene mutation analysis in patients affected by breast, melanoma, pancreatic, prostate or ovarian tumors. PVs: pathogenic variants; CIP: conflicting interpretations of pathogenicity; VUS: variants of uncertain significance; WT: wild-type *BRCA1/2* sequence.

A total of 1027 patients were screened for germline *BRCA1/2* mutations between January 2017 and May 2024. Molecular analyses were performed at the Center of Experimental Oncology and Hematology of the Hospital Policlinico “G. Rodolico—San Marco” of Catania, according to Sicilian guidelines (http://www.gurs.regione.sicilia.it/Indicep1.htm, accessed on 11 July 2024). Among the 1027 recruited, 860 subjects had breast cancer, 48 had ovarian cancer, 60 had pancreatic cancer, 47 had prostate cancer and 12 had melanomas. Patient distribution according to cancer type and data results is shown in Figure 1.

We selectively focused our study on the breast cancer cohort. These patients displayed a median age of 55 years (interquartile range of 47–65) and were mainly females (*n* = 831, 97%).

**Figure 2:**
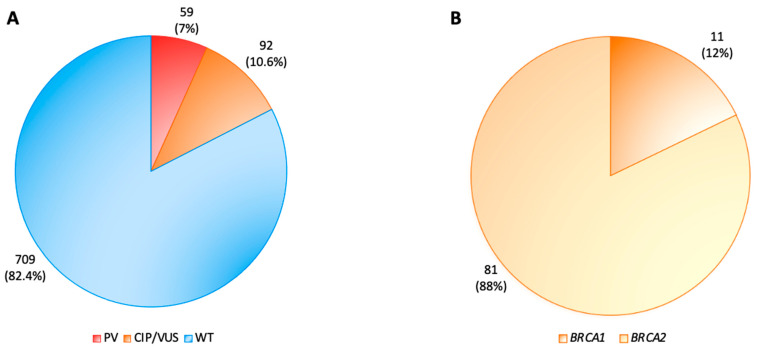
*BRCA1* and *BRCA2* mutation analysis of 860 breast cancer patients. (**A**) Distribution of pathogenic variants (PVs) (red), conflicting interpretations of pathogenicity (CIP) or variants of uncertain significance (VUS) (orange) and WT (blue) in 860 patients with breast cancer; (**B**) 92 (10.6%) out 860 subjects harbored CIP or VUS, 11 (12%) in the *BRCA1* gene (dark orange) and 81 (88%) in the *BRCA2* gene (light orange). PVs: pathogenic variants; CIP: conflicting interpretations of pathogenicity; VUS: variants of uncertain significance; WT: wild-type *BRCA1/2* sequence.

Among these subjects, 151 (18%) harbored a *BRCA1/2* mutation, and only one patient was male, whereas all of the others were women. Fifty-nine (7%) had a PV, while ninety-two (10.6%) harbored a CIP/VUS. Moreover, 29 of the 58 pathogenic variants (49.2%) occurred in *BRCA1,* and 30 (50.8%) occurred in *BRCA2*, while 11 CIP/VUSs were in *BRCA1* (12%) and 81 (88%) were in *BRCA2* (Figure 1 and Figure 2). No large genomic rearrangements were detected by MLPA analysis.

### 3.2. Description and Localization of BRCA1 and BRCA2 CIP/VUS Variants

Next, we evaluated the type of *BRCA1* and *BRCA2* CIP/VUS variants. Among the ninety-two patients harboring CIP/VUS, two subjects presented the same alteration (c.5333A > G) in the *BRCA1* sequence; therefore, we found ten different *BRCA1* CIP/VUS alterations. Nine of these alterations were single nucleotide variants (SNVs), whereas only one was a deletion (c.4063_4065delAAT) (Table 1).

Concerning *BRCA2*, the genetic analysis showed a total of 55 different CIP/VUS mutations in 81 individuals. Furthermore, it is of interest that the c.3509C > T alteration was found in five subjects, the c.7871A > G mutation was found in seven patients, four individuals presented the c.8850G > T variation, the c.9586A > G variant was found in three patients and the c.9613_9614delGCinsCT del/ins was found in three subjects. Each of the other eight alterations (c.28A > G, c.442T > C, c.599C > T, c.1054T > C, c.3070A > G, c.4769A > G, c.5870T > C and c.9698G > A) were attained in two individuals. Moreover, in the case of the *BRCA2* sequence, fifty-three of fifty-five alterations were SNVs, while only one was a deletion (c.9052_9057delAGTAAA) and another one a deletion/insertion (c.9613_9614delGCinsCT) (Table 2). Specifically, the c.7871A > G alteration at the protein level, found in the majority of our patients, results in the change of a tyrosine to a cysteine located in the helical domain. It is of interest that the *BRCA2* c.9052_9057delAGTAAA alteration deletes six nucleotides from exon 23 of the *BRCA2* mRNA, and it is predicted to result in an in-frame deletion. Furthermore, the c.9613_9614delGCinsCT alteration replaces alanine with leucine at the codon 3205 of the BRCA2 protein. The alanine amino acid is moderately conserved, and it has similar physiochemical characteristic to leucine.

**Figure 3:**
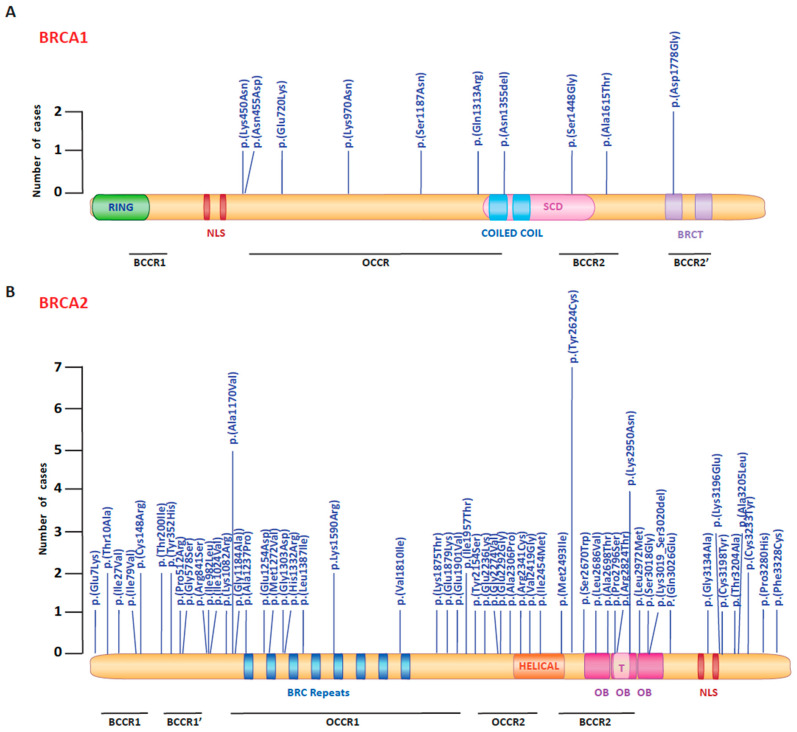
Representations of BRCA1 and BRCA2 proteins and localization of conflicting interpretations of pathogenicity or variants of uncertain significance. The figure shows the distribution of *BRCA1* (**A**) and *BRCA2* (**B**) CIP/VUS amino acid changes in breast cancer patients. The bar heights show the number of cases. BRCA1 and BRCA2 proteins are painted with their functional domains. (**A**) The BRCA1 protein includes a RING domain (RING) and a nuclear localization sequence (NLS), a coiled-coil domain, an SQ/TQ cluster domain (SCD) and BRCA1 C-terminus domains (BRCT). (**B**) The BRCA2 protein contains 8 BRC repeats, a DNA binding domain with a helical domain (Helical), a 3 oligonucleotide/oligosaccharide binding (OB) fold, a tower domain (T) and an NLS at the C-terminus. Regions referred to as breast cancer cluster regions (BCCRs) and ovarian cancer cluster regions (OCCRs) are indicated at the bottom of the figure. CIP = conflicting interpretations of pathogenicity; VUS = variants of uncertain significance.

Subsequently, we mapped the *BRCA1* and *BRCA2* CIP/VUS alterations through the proteins’ binding regions and functional domain (Figure 3). In the *BRCA1* gene, the 10 CIP/VUS variants were distributed along the entire gene sequence. Among these, the c.4063_4065delAAT deletion was located inside the coiled-coil domain, which binds to the WD 40 domain of the *PALB2* gene. Among the *BRCA2* gene, 30.1% variants were located in the BCCR regions and 36.4% of CIP/VUS alterations in the OCCRs. For BRCA2 protein, we found that two CIP/VUS alterations (c.7479G > A, c.7871G > A) were mapped in the DNA Binding Domain.

### 3.3. In Silico Evaluation of BRCA1 and BRCA2 CIP/VUS Variants

**Figure 4:**
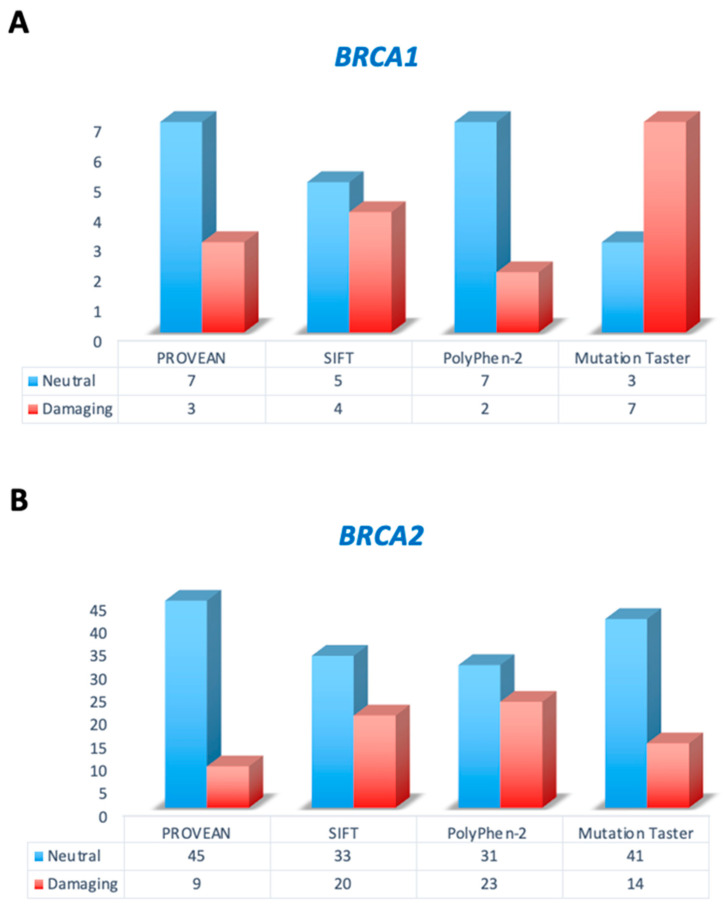
Distribution of *in silico* prediction outcomes on *BRCA1* and *BRCA2* CIP/VUS alterations. The histograms show the distribution of CIP/VUS alterations in *BRCA1* (**A**) and *BRCA2* (**B**) genes predicted as neutral (light blue) and damaging (light red) by PROVEAN, SIFT, PoliPhen-2 and MT. The number reported inside the box indicated the number of CIP/VUS alterations obtained by each *in silico* tools analysis. CIP = conflicting interpretations of pathogenicity; MT = Mutation Taster; PROVEAN = Protein Variation Effect Analyzer; SIFT = Sorting Intolerant From Tolerant and VUS = variants of uncertain significance.

In order to investigate the prediction of the effects and potential clinical significance of *BRCA1* and *BRCA2* CIP/VUS variants, we compared the performance of four different *in silico* tools. Each missense alteration was analyzed with the PROVEAN, SIFT, PolyPhen-2 and Mutation Taster, whereas the *BRCA1* deletion CIP/VUS mutation (c.4063_4065delAAT) was interrogated with the PROVEAN and Mutation Taster and the only *BRCA2* del/ins (c.9613_9614delGCinsCT) were interrogated with the PolyPhen-2 and Mutation Taster (Table 1). The *in silico* analysis for *BRCA1* CIP/VUS alterations predicted it as reaching a 3/10 (30%) damaging alteration of the variant with either PROVEAN or Mutation Taster, 4/9 (44.4%) with SIFT and 7/9 (77.8%) with PolyPhen-2 (Figure 4A). Among *BRCA2* CIP/VUS variations, the *in silico* prediction achieved a 9/54 (16.7%) damaging alteration with PROVEAN, 20/53 (37.8%) with SIFT, 23/54 (42.6%) with PolyPhen-2 and 14/55 (34.1%) with Mutation Taster (Figure 4B).

**Figure 5:**
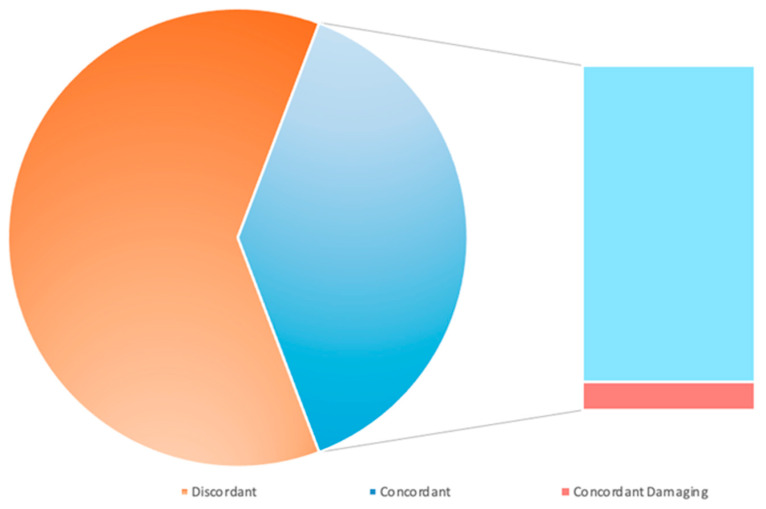
Concordance *in silico* prediction outcomes for *BRCA2* CIP/VUS variants. Distribution of the discordant (light orange) and concordant (blue) *BRCA2* CIP/VUS variations. Among the concordant alterations, 92% are predicted as neutral (light blue), whereas 8% are predicted as damaging (light red). CIP = conflicting interpretations of pathogenicity; VUS = variants of uncertain significance.

Next, we wanted to evaluate the agreement rate of the four *in silico* tools. To this end, we focused this analysis only on the 55 *BRCA2* CIP/VUS alterations, due to the larger sample size. The analysis of all CIP/VUS variants by the four approaches showed that 42/55 alterations were predicted as damaging at least one *in silico* used tool. Prediction agreement was achieved in 25/55 (45.5%) CIP/VUS mutations, with 23 (92%) and 2 (8%) CIP/VUS alterations predicted as neutral and damaging, respectively (Figure 5).

**Figure 6:**
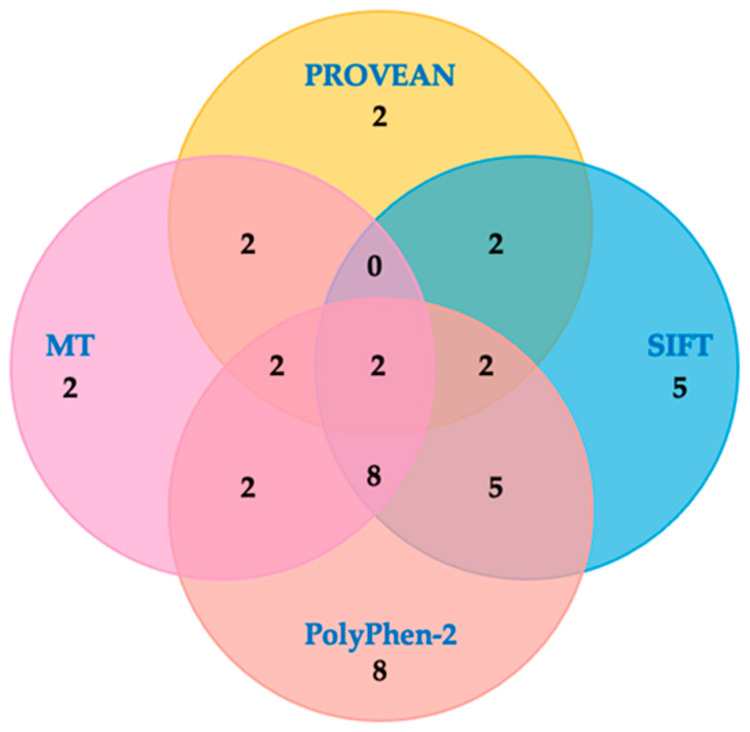
Concurrence in prediction outcomes between PROVEAN, SIFT, PoliPhen-2 and MT for *BRCA2* CIP/VUS alterations. Venn diagram represents the *in silico* prediction on 42 CIP/VUS alterations with predicted damaging effects. Variants were interrogated by PROVEAN (light yellow), SIFT (light blue), PoliPhen-2 (light red) and MT (light pink). Number inside the circle indicated the number of alterations with an agreement prediction effect. CIP = conflicting interpretations of pathogenicity; MT = Mutation Taster; PROVEAN = Protein Variation Effect Analyzer; SIFT = Sorting Intolerant From Tolerant; VUS = variants of uncertain significance.

Lasty, we assessed the consensus in prediction outcomes for the 42 tested CIP/VUS alterations with an estimated damaging effect. We found a concordance for all four *in silico* tools in two alterations (c.7871A > G, c.8939C > A) (Figure 6). Moreover, the highest consensus was obtained in 12/42 (28.6%) mutations by considering three out four *in silico* tools. In detail, a concordance between SIFT, PolyPhen-2 and MT was observed for eight variants, whereas two CIP/VUS alterations were in agreement with PROVEAN, PolyPhen-2 and MT, or PROVEAN, SIFT and PolyPhen-2. Notably, 17/42 (40.5%) of the predicted variants were unique for being damaging in *in silico* tools alone (Figure 6).

## 4. Discussion

Accurate classification of molecular alterations with regard to their pathogenic potential is of pivotal importance, especially for patients presenting a familial cancer history. Recently, the development of high-throughput technologies determined unprecedented progress in *BRCA1* and *BRCA2* genetic testing. To this aim, the molecular analysis of *BRCA1* and *BRCA2* allows the identification of patients with high probability to develop different tumor types, including breast, ovarian and prostate cancer, among others. At the present time, approximately 20.000 *BRCA1/2* variants have been recognized and categorized according to the American College of Medical Genetics [29] and ENIGMA systems [7,30,31]. With the recent increase in the use of NGS technology, the spectrum of missense and spicing alteration, described as CIP and VUS alterations, were increased in the *BRCA1* and *BRCA2* sequence by a genetic test. It was estimated that up to 15% of VUSs were identified among European subjects, with higher rates in African-American and Hispanic populations [15,16,32]. To this regard, the useful approaches that measure the impact of CIP or VUS on biological processes represent an alternative method for pathogenic prediction and clinical annotations.

In this study, we used four *in silico* prediction tools in order to predict the effect and potential significance of the *BRCA1* and *BRCA2* CIP/VUS alterations for the patient’s clinical management. First, we characterized the CIP/VUS alterations according to the ENIGMA and ClinVar databases. Of the 860 BC patients analyzed, 10.6% harbored *BRCA1* and *BRCA2* CIP/VUS mutations. This frequency was concordant with previous data reported in the literature [16]. The highest numbers of CIP/VUS were distributed in the *BRCA2* sequence, and similar findings were observed in published studies [33,34,35]. Our analysis showed a CIP/VUS landscape that is differently distributed in the studied population. Indeed, while some CIP/VUSs were detected only in a single subject, others were repeated in more individuals, such as the c.3509C > T and c.7871A > G, which were observed in two different groups of seven patients. For c.3509C > T, although this variant is still defined as a CIP mutation, several findings, including co-occurrence, have a lack of segregation with the disease of association in case-control studies, thus supporting its classification as benign [36,37,38,39]. Moreover, it occurs at a poorly conserved position in BRCA protein. Concerning the c.7871A > G alteration, it was previously described in a cohort of breast and ovarian cancer. At the protein level, it causes the substitution of a conservative amino acid that results in a reduced stability of BRCA2 protein [40,41]. Furthermore, two del/ins were identified in the *BRCA2* gene. The first is represented by the deletion of c.9052_9057delAGTAAA, observed in a subject and previously reported in individuals with personal or family history of cancer related to *BRCA* [42]. However, to date, its clinical significance remains unclear. The second del/ins is the c.9613_9614delGCinsCTdel/ins, also previously detected in both breast and prostate cancer subjects [43,44,45]. This variant rises at a non-conserved position and in a domain of unknown function.

Subsequently, we mapped the *BRCA1* and *BRCA2* CIP/VUS alterations along the functional domains of the protein and the gene putative OCCR and BCCR regions, which were previously defined by Rebbeck et al. [46]. These regions were defined as dangerous for developing breast and ovarian cancer, respectively. With regards to the gene localization of CIP/VUS, the c.4063_4065delAAT deletion interested the coiled-coil domain of the BRCA1 protein. This domain is crucial for the interaction with PALB2 that binds to BRCA2 and acts by recruiting BRCA2 to the chromatin and promoting the homologous recombination. Loss of this interaction results in an increase of tumorigenesis [47]. The *BRCA2* CIP/VUSs were distributed along the entire sequence. Among them, the c.7479G > A alteration is mapped in the DNA Binding Domain (DBD), as required for homologous recombination [48]. This domain is located in the C-terminal region encompassed Helical domain, Tower domain and three OB folds. The DBD and RAD51 interacting sites of BRCA2 are able to promote the assembly of RAD51 toward single-strand DNA and single-strand DNA/double-strand DNA connection [49].

To understand whether the CIP/VUS variants may potentially be damaging, we interrogated the four *in silico* prediction tools, PROVEAN, SIFT, PolyPhen-2 and Mutation Taster. Since CIP/VUSs are mostly a change of amino acid with residues with the same properties or in-frame ins/del alteration, their impact on protein function is often complex for the interpreter. The *in silico* interrogation is predicted as damaging few of the CIP/VUS alterations. Although a similar performance was observed, the PolyPhen-2 and SIFT tools were mostly predicted to damage alterations (47.6% and 39%), compared to the other two *in silico* approaches (MT = 26.1% and PROVEAN = 19%). Interestingly, both the c.7871A > G and c.9839C > A mutations were predicted to have a damaging effect by the four algorithms. The c.7871A > G alteration has been described in several breast and ovarian cancer patients. This mutation mapped at codon 2624 of the BRCA2 protein (p.Tyr2624Cys) in the helical domain. This domain binds the 70 amino acids deleted in split-end/split food syndrome (DSS1) protein. This variation results in a significant decrease in structural stability, suggesting a damaging effect on protein function [41]. To date, supporting evidence on its function is conflicting, because the clinical significance of this alteration is still unclear [40,50,51]. Furthermore, the c.9839C > A alteration, located in exon 26 of the *BRCA2* sequence, has been shown to modify a poorly conserved amino acid. Its clinical interpretation is still controversial, and a functional test suggested its neutral effect on protein function, whereas *in silico* approaches predicted a damaging impact, as previously reported [31,52,53].

A limitation of the current study was the small size of the dataset of CIP/VUS alterations detected in the *BRCA1* sequence. Consequently, we focused our next analysis on *BRCA2* CIP/VUS mutations. When we considered the four approaches to predict the effect of the variants on the clinical outcome, a low consensus was achieved for the CIP/VUSs classified as damaging (8%), compared to neutral. The absence of agreement between the *in silico* tools can generate confusion in the interpretation of these variants. To this regard, there are studies with controversial results. Some of them suggested that the combination of different *in silico* algorithms may help to improve the prediction performance. Other researchers reported opposite data [17,54,55]. In our study, a higher concordance was obtained (28.6%) when we used three of four *in silico* tools, suggesting that this approach may be more efficient than the previous one.

## 5. Conclusions

The inherent uncertainty related to the nature of CIP/VUS does not allow us to classify these alterations as deleterious or benign, potentially influencing patients’ management. In this context, the use of bioinformatic *in silico* tools may help to identify variants with a potentially damaging effect. Still, the lack of a substantial agreement between the different algorithms suggests that these bioinformatic approaches should be combined with an accurate assessment of the family and clinical history of the proband.

## Figures and Tables

**Table 1:** *BRCA1* gene variants with conflicting clinical interpretations of pathogenicity.

Nucleotide Change HGVS Nomenclature	Amino Acid Change	Type of Alteration	ENIGMA/ ClinVar Classification	PROVEAN	SIFT	PolyPhen-2	Mutation Taster	No. of Patients
c.1350A > T	p. (Lys450Asn)	Missense	NYR/CIP	Damaging	Damaging	Damaging	Neutral	1
c.1363A > G	p. (Asn455Asp)	Missense	NYR/CIP	Damaging	Damaging	Neutral	Neutral	1
c.2158G > A	p. (Glu720Lys)	Missense	NYR/CIP	Damaging	Neutral	Damaging	Damaging	1
c.2910A > C	p. (Lys970Asn)	Missense	NYR/CIP	Neutral	Neutral	Damaging	Neutral	1
c.3560G > A	p. (Ser1187Asn)	Missense	NYR/CIP	Neutral	Neutral	Damaging	Neutral	1
c.3938A > G	p. (Gln1313Arg)	Missense	NYR/CIP	Neutral	Neutral	Damaging	Neutral	1
c.4063_4065delAAT	p. (Asn1355del)	Deletion	NYR/CIP	Neutral	NI	NI	Neutral	1
c.4342A > G	p. (Ser1448Gly)	Missense	NYR/CIP	Neutral	Neutral	Neutral	Damaging	1
c.4843G > A	p. (Ala1615Thr)	Missense	NYR/VUS	Neutral	Damaging	Damaging	Neutral	1
c.5333A > G	p. (Asp1778Gly)	Missense	NYR/CIP	Neutral	Damaging	Damaging	Damaging	2

CIP: conflicting interpretation of pathogenicity; DEL: deletion; ENIGMA: evidence-based network for the interpretation of germline mutant alleles; HGVS: human genome variant society; NI: not investigated; NYR: not yet reviewed; VUS: variant of uncertain significance.

**Table 2:** *BRCA2* gene variants with conflicting clinical interpretations of pathogenicity.

Nucleotide Change HGVS Nomenclature	Amino Acid Change	Type of Alteration	ENIGMA/ ClinVar Classification	PROVEAN	SIFT	PolyPhen-2	Mutation Taster	No. of Patients
c.19G > A	p. (Glu7Lys)	Missense	NYR/CIP	Neutral	Neutral	Neutral	Neutral	1
c.28A > G	p. (Thr10Ala)	Missense	NYR/CIP	Neutral	Damaging	Neutral	Neutral	2
c.79A > G	p. (Ile27Val)	Missense	NYR/CIP	Neutral	Neutral	Neutral	Neutral	1
c.235A > G	p. (Ile79Val)	Missense	NYR/CIP	Neutral	Neutral	Neutral	Neutral	1
c.442T > C	p. (Cys148Arg)	Missense	NYR/CIP	Neutral	Neutral	Neutral	Neutral	2
c.599C > T	p. (Thr200Ile)	Missense	NYR/CIP	Neutral	Damaging	Neutral	Neutral	2
c.1054T > C	p. (Tyr352His)	Missense	NYR/CIP	Neutral	Neutral	Neutral	Neutral	2
c.1535C > G	p. (Pro512Arg)	Missense	NYR/CIP	Neutral	Neutral	Damaging	Neutral	1
c.1732G > A	p. (Gly578Ser)	Missense	NYR/CIP	Neutral	Damaging	Neutral	Neutral	1
c.2523A > T	p. (Arg841Ser)	Missense	NYR/VUS	Neutral	Neutral	Neutral	Neutral	1
c.2944A > C	p. (Ile982Leu)	Missense	NYR/CIP	Neutral	Neutral	Neutral	Neutral	1
c.3070A > G	p. (Ile1024Val)	Missense	NYR/CIP	Neutral	Neutral	Neutral	Neutral	2
c.3245A > G	p. (Lys1082Arg)	Missense	NYR/CIP	Neutral	Neutral	Neutral	Neutral	1
c.3509C > T	p. (Ala1170Val)	Missense	NYR/CIP	Neutral	Neutral	Neutral	Neutral	5
c.3551G > C	p. (Gly1184Ala)	Missense	NYR/CIP	Neutral	Neutral	Neutral	Neutral	1
c.3709G > C	p. (Ala1237Pro)	Missense	NYR/CIP	Damaging	Neutral	Damaging	Damaging	1
c.3762G > T	p. (Glu1254Asp)	Missense	NYR/CIP	Neutral	Neutral	Damaging	Neutral	1
c.3814A > G	p. (Met1272Val)	Missense	NYR/CIP	Neutral	Neutral	Neutral	Neutral	1
c.3908G > A	p. (Gly1303Asp)	Missense	NYR/CIP	Damaging	Neutral	Neutral	Neutral	1
c.3995A > G	p. (His1332Arg)	Missense	NYR/CIP	Neutral	Neutral	Neutral	Neutral	1
c.4159T > A	p. (Leu1387Ile)	Missense	NYR/CIP	Neutral	Damaging	Damaging	Neutral	1
c.4769A > G	p. (Lys1590Arg)	Missense	NYR/CIP	Neutral	Damaging	Damaging	Neutral	2
c.5428G > A	p. (Val1810Ile)	Missense	NYR/CIP	Neutral	Damaging	Neutral	Neutral	1
c.5624A > C	p. (Lys1875Thr)	Missense	NYR/CIP	Damaging	Neutral	Damaging	Neutral	1
c.5635G > A	p. (Glu1879Lys)	Missense	NYR/CIP	Damaging	Neutral	Neutral	Neutral	1
c.5702A > T	p. (Glu1901Val)	Missense	NYR/CIP	Damaging	Damaging	Damaging	Neutral	1
c.5870T > C	p. (Ile1957Thr)	Missense	NYR/CIP	Neutral	Neutral	Neutral	Neutral	2
c.6461A > C	p. (Tyr2154Ser)	Missense	NYR/CIP	Neutral	Neutral	Neutral	Neutral	1
c.6706G > A	p. (Glu2236Lys)	Missense	NYR/CIP	Neutral	Damaging	Damaging	Damaging	1
c.6821G > T	p. (Gly2274Val)	Missense	NYR/CIP	Neutral	Neutral	Damaging	Damaging	1
c.6875A > G	p. (Glu2292Gly)	Missense	NYR/VUS	Neutral	Damaging	Damaging	Damaging	1
c.6916G > C	p. (Ala2306Pro)	Missense	NYR/CIP	Neutral	Neutral	Neutral	Neutral	1
c.7021C > T	p. (Arg2341Cys)	Missense	NYR/CIP	Neutral	Neutral	Damaging	Neutral	1
c.7256T > G	p. (Val2419Gly)	Missense	NYR/CIP	Neutral	Neutral	Neutral	Neutral	1
c.7362T > G	p. (Ile2454Met)	Missense	NYR/VUS	Neutral	Damaging	Damaging	Neutral	1
c.7479G > A	p. (Met2493Ile)	Missense	NYR/VUS	Neutral	Neutral	Neutral	Damaging	1
c.7871A > G	p. (Tyr2624Cys)	Missense	NYR/CIP	Damaging	Damaging	Damaging	Damaging	7
c.8009C > G	p. (Ser2670Trp)	Missense	NYR/CIP	Neutral	Damaging	Damaging	Damaging	1
c.8056C > G	p. (Leu2686Val)	Missense	NYR/CIP	Neutral	Damaging	Damaging	Damaging	1
c.8092G > A	p. (Ala2698Thr)	Missense	NYR/CIP	Neutral	Neutral	Neutral	Neutral	1
c.8386C > T	p. (Pro2796Ser)	Missense	NYR/CIP	Neutral	Neutral	Neutral	Neutral	1
c.8471G > C	p. (Arg2824Thr)	Missense	NYR/CIP	Neutral	Damaging	Damaging	Damaging	1
c.8850G > T	p. (Lys2950Asn)	Missense	NYR/CIP	Neutral	Damaging	Damaging	Damaging	4
c.8914T > A	p. (Leu2972Met)	Missense	NYR/CIP	Neutral	Neutral	Damaging	Damaging	1
c.9052A > G	p. (Ser3018Gly)	Missense	NYR/CIP	Neutral	Neutral	Neutral	Neutral	1
c.9052_9057delAGTAAA	p. (Lys3019_Ser3020del)	Deletion	NYR/CIP	Damaging	NI	NI	Damaging	1
c.9076C > G	p. (Gln3026Glu)	Missense	NYR/CIP	Neutral	Damaging	Damaging	Damaging	1
c.9401G > C	p. (Gly3134Ala)	Missense	NYR/VUS	Neutral	Damaging	Neutral	Neutral	1
c.9586A > G	p. (Lys3196Glu)	Missense	NYR/CIP	Neutral	Damaging	Damaging	Neutral	3
c.9593G > A	p. (Cys3198Tyr)	Missense	NYR/CIP	Neutral	Neutral	Neutral	Neutral	1
c.9610A > G	p. (Thr3204Ala)	Missense	NYR/VUS	Neutral	Neutral	Neutral	Neutral	1
c.9613_9614delGCinsCT	p. (Ala3205Leu)	Missense	NYR/CIP	NI	NI	Damaging	Neutral	3
c.9698G > A	p. (Cys3233Tyr)	Missense	NYR/CIP	Damaging	Damaging	Neutral	Neutral	2
c.9839C > A	p. (Pro3280His)	Missense	NYR/CIP	Damaging	Damaging	Damaging	Damaging	1
c.9983T > G	p. (Phe3328Cys)	Missense	NYR/CIP	Neutral	Neutral	Damaging	Neutral	1

CIP: conflicting interpretation of pathogenicity; DEL: deletion; ENIGMA: evidence-based network for the interpretation of germline mutant alleles; HGVS: human genome variant society; NI: not investigated; NYR: not yet reviewed; VUS: variant of uncertain significance.

## Data Availability

The original contributions presented in the study are included in the article, further inquiries can be directed to the corresponding author.
